# Periprocedural Safety of Interventional Electrophysiological Procedures in Octogenarians and Nonagenarians

**DOI:** 10.1111/jce.16689

**Published:** 2025-05-26

**Authors:** Vanessa Sciacca, Jakob Feldt, Laura Rottner, Christian‐Hendrik Heeger, Christian Sohns, Bruno Reissmann, Ardan M. Saguner, Francesco Santoro, Roland R. Tilz, Tilman Maurer, Andreas Rillig, Feifan Ouyang, Dominik Linz, Kevin Vernooy, Philipp Sommer, Arian Sultan, Stephan Willems, Karl‐Heinz Kuck, Andreas Metzner, Thomas Fink

**Affiliations:** ^1^ Department of Cardiology Asklepios Klinik St. Georg Hamburg Germany; ^2^ Clinic for Electrophysiology Herz‐ und Diabeteszentrum NRW Bad Oeynhausen Germany; ^3^ Department of Cardiac Electrophysiology University Heart Center, University Hospital Hamburg Eppendorf Hamburg Germany; ^4^ Department of Cardiac Electrophysiology University Hospital Schleswig‐Holstein–Campus Lübeck Lübeck Germany; ^5^ Department of Cardiology University Heart Center, University Hospital Zurich Zurich Switzerland; ^6^ Department of Medical and Surgical Sciences University of Foggia Foggia Italy; ^7^ Department of Cardiology Unit University Polyclinic Hospital of Foggia Foggia Italy; ^8^ Department of Cardiology, Cardiovascular Research Institute Maastricht (CARIM) Maastricht University Medical Center Maastricht The Netherlands; ^9^ Department of Biomedical Sciences, Faculty of Health and Medical Sciences University of Copenhagen Copenhagen Denmark; ^10^ Centre for Heart Rhythm Disorders University of Adelaide, Royal Adelaide Hospital Adelaide Australia

**Keywords:** catheter ablation, complications, elderly, LAA closure, risk stratification

## Abstract

**Background:**

Catheter ablation is an established treatment for cardiac arrhythmia. There is a lack of data on invasive electrophysiological (EP) procedures in aged patients.

**Methods:**

Consecutive patients ≥ 80 years who underwent catheter ablation or left atrial appendage closure procedures between January 2005 and December 2017 in a high‐volume center were retrospectively studied and compared to a matched control group of individuals < 80 years of age.

**Results:**

The aged group consisted of 486 patients who underwent 566 procedures at a mean age of 82.7 ± 2.5 years (range 80−95 years). A cohort of 480 patients aged < 80 years (mean age 64.1 ± 13.3 years) with 566 procedures served as a control group. Performed procedures were atrial arrhythmia ablation including atrial fibrillation treatment (*n* = 366, 64.7%), cavotricuspid isthmus ablation (*n* = 139, 24.6%), ablation of ventricular arrhythmias (*n* = 57, 10.1%), and left atrial appendage closure (*n* = 12, 2.1%). There were numerically more procedures with major complications after treatment of elderly patients (32 [5.7%] vs. 21 [3.5%] procedures, *p* = 0.12), as well as numerically more procedures accompanied by intrahospital deaths (6 [1.1%] vs. 1 [0.2%] procedure, *p* = 0.12). The rate of minor complications was significantly higher in aged patients as compared to younger controls (31 [5.1%] vs. 17 [20%] procedures, *p* = 0.039).

**Conclusion:**

Invasive EP procedures in octogenarians and nonagenarians are feasible, however a significantly higher incidence of minor periprocedural complications and a trend toward more severe complications and intrahospital fatalities were observed compared to younger patients. These findings support an individual risk‐benefit assessment for elderly individuals before invasive EP treatments are conducted.

## 1 Introduction

Electrophysiological (EP) procedures have become routine treatment for patients with atrial and ventricular dysrhythmias over the past 20 years. Procedures such as diagnostic EP studies, catheter ablation of atrial fibrillation (AF), atrial tachycardias (AT), the cavo‐tricuspid isthmus (CTI), supraventricular tachycardias (SVT), or ventricular tachycardias (VT) and percutaneous left atrial appendage closure (LAAC) procedures are considered to be safe and effective and found implementation in current guidelines, based on results of numerous studies and registries [1, 2]. Nevertheless, efficacy and safety of catheter ablation strongly depend on the underlying type of arrhythmia being treated and may be influenced by patient characteristics such as comorbidity, hereditary factors, and age. The majority of conducted studies were derived from patients of younger age and therefore conclusions on efficacy and safety cannot be transferred to aged patients. Until today, there is limited data on aged patients especially for ablation procedures for AF and ventricular arrhythmias. This gap in scientific evidence is reflected by the results of a European Heart Rhythm Association (EHRA) survey, which reported that many European centers have an age limit of 75−85 years with regard to perform catheter ablation for atrial and ventricular arrhythmias [3]. This study sought to investigate the periprocedural safety and efficacy of elective EP procedures in a collective of aged patients in a high‐volume ablation center.

## 2 Methods

### 2.1 Study Design and Patient Inclusion

This retrospective study included consecutive patients undergoing electrophysiologic procedures from January 2005 until December 2017 in Asklepios Klinik St. Georg, Hamburg, Germany. Procedures included all types of invasive diagnostic EP studies as well as catheter ablation procedures of AF, SVT, AT and VT, and LAA closure procedures. Patients with an age ≥ 80 years were included in the study and compared with a younger control population. The analysis was approved by the local ethics committee (processing number: WF‐48/17). All patients included in the analysis gave written informed consent for the ablation procedure and patient information was anonymized for analysis. The patient cohort consisted of patients with idiopathic VA and of patients with structural heart disease. Procedural indications were based on corresponding guidelines.

### 2.2 Procedural Setup

Procedures were conducted under deep sedation using fentanyl or sufentanil, midazolam, and propofol. Procedures were performed under general anesthesia with endotracheal intubation only in patients with decompensated heart failure or impaired oxygenation. Transthoracic echocardiography was performed before the procedure to assess left ventricular ejection fraction (LV‐EF) and to rule out left ventricular (LV) thrombi in patients without known AF and in case of known structural heart disease. Transesophageal echocardiography (TEE) was performed in patients with known AF. In case of transseptal puncture or LV access, intravenous heparin was administered targeting an activated clotting time (ACT) > 300 s. In cases of right heart procedures, a prophylactic dosage of heparin (3000−5000 IE) was administered. In patients with pre‐existing treatment with vitamin K antagonists, the procedure was performed on bridging therapy with low‐molecular heparin (LMH) until 2012. Afterward, no bridging was performed and vitamin K antagonists were not interrupted aiming therapeutic INR values at the day of the procedure. Direct oral anticoagulant (DOAC) therapy was discontinued 24 h before the procedure. Anti‐arrhythmic drug therapy (AAD), except amiodarone treatment, was discontinued at least 5 half‐times before the procedure.

### 2.3 EP Study, Electroanatomic Mapping (EAM), and Ablation

Vascular access was gained at the femoral veins, arteries, and/or subclavian access depending on procedure types, patient's anatomy, and operator's preference. For diagnostic EP studies, SVT and AF/AT ablation procedures diagnostic catheters were positioned in the coronary sinus (CS), at the His bundle and the right ventricle (RV). Afterward, programmed stimulation was performed. The stimulation protocol consisted of programmed stimulation from the RV at least at two drive cycle lengths (CL; 510 and 440 ms) with up to three extra stimuli to a minimum coupling interval of 200 ms. Burst‐pacing with the shortest CL of 250 ms was also used if induction failed with programmed stimulation.

EAM was performed using commercially available 7 F 3.5 mm tip ablation catheters (Celsius or Thermocool, Biosense Webster Inc., Diamond Bar, CA, USA) through the femoral vein under fluoroscopic guidance. For 3D EAM, a point‐by‐point map was acquired including activation mapping in cases of organized atrial or ventricular arrhythmias. Nonirrigated or irrigated radio‐frequency current was delivered in power‐controlled mode, depending on the underlying arrhythmia and on the operators' preference. In case of left atrial (LA) or LV ablation, irrigated ablation was mandatory. For AF ablation, ablation approaches used during the study period included pulmonary vein isolation (PVI) as the cornerstone of the procedures as well as left and right atrial linear ablation, focal ablation, or ablation of complex fractionated atrial electrograms. Ablation of VT was performed with endocardial and/or epicardial approaches for mapping and ablation using ante‐ and/or retrograde access. LAAC was performed with an endocardial device (Watchman, Boston Scientific, Marlborough, MA, USA). For LA and LV access transseptal puncture was performed under fluoroscopic guidance with a modified Brockenbrough approach.

### 2.4 Postprocedural Care and in‐Hospital Follow‐Up

All patients underwent transthoracic echocardiography at the end of the procedure and the day after the procedure to rule out pericardial effusion. After epicardial mapping/ablation, an epicardial pigtail catheter was inserted at the end of the procedure and kept for at least 6 h. The catheter was removed after exclusion of pericardial effusion. A chest X‐ray to rule out pneumothorax was performed in patients with attempted subclavian vein access. Pre‐existing therapy with vitamin K antagonists was continued aiming at an INR of 2.0−3.0. If there was no major bleeding 6 h following the procedure, DOACs were restarted. Postprocedural in‐hospital ablation success was evaluated with Holter ECG recordings at the first postprocedural day, daily 12‐lead ECG recordings, interrogations of intracardiac devices (if available), and continuous telemetry monitoring on hospital wards in patients with sustained VA.

### 2.5 Definition of Periprocedural Complications and Complication Analysis

Periprocedural complications were defined as major when life‐threatening or resulting in patient death, permanent or temporal patient disability, leading to percutaneous or surgical intervention, leading to transfusion of blood products, or leading to prolonged hospitalization. Other complications which resulted in discomfort or pain or further diagnostic procedures were categorized as minor complications. All complications occurring during the hospital stay were analyzed. A cohort of patients aged < 80 years, who underwent catheter ablation between 2005 and 2017 served as a control group to compare frequencies of major adverse events. Procedures of the study and control groups were manually matched according to the type of EP procedures performed and the timepoint of procedures. For each procedure of the aged group, the following procedure of the same type in an individual aged < 80 years which was scheduled in our center, was analyzed.

### 2.6 Statistics

Continuous variables were expressed as mean ± standard deviation (SD) for normal distributions or as median/interquartile range (IQR) for non‐normal distributions or categorical data. Categorial variables were displayed as counts (%). Logistic regression for prediction of complications (major complications or death and minor complications) was performed. Effects were displayed as log odds plots. Three outcomes were assessed (no complications or minor complication or major complication/death). Two‐sided *p* < 0.05 were considered significant. All analyses were performed using SPPS version 25 (IBM Cooperation, Armonk, New York) or R (R Foundation, Vienna, Austria).

## 3 Results

From January 2005 to December 2017, a total of 24 448 diagnostic and therapeutic EP procedures and 458 LAAC procedures were performed in the EP laboratories of the Asklepios Klinik Hamburg St. Georg (Supporting Information S1: Table 1).

**Table 1:** Baseline data of the aged and younger groups.

	Aged group	Younger group	
Parameter	*n* (%) or mean ± SD	*n* (%) or mean ± SD	*p* value
Patients (*n*)	486	480	
Procedures (*n*)	566	566	
Mean age (years)	82.7 ± 2.5	64.1 ± 13.3	*< 0.001*
Male, *n* (% of procedures)	252 (44.5)	358 (63.3)	*< 0.001*
Mean ejection fraction (%)	56.7 ± 13.6	53.9 ± 11.2	*< 0.001*
EF < 50%, *n* (% of procedures)	31 (5.5)	92 (16.3)	*< 0.001*
EF < 30%, *n* (% of procedures)	12 (2.1)	13 (2.3)	1.0
Previous myocardial infarction, *n* (% of procedures)	56 (9.9)	76 (13.4)	0.064
Arterial hypertension, *n* (% of procedures)	415 (73.3)	388 (68.6)	0.077
Diabetes mellitus, *n* (% of procedures)	70 (12.4)	72 (12.7)	0.86
Kidney failure, *n* (% of procedures)	129 (22.8)	36 (6.3)	*< 0.001*
Previous stroke or transient ischemic attack, *n* (% of procedures)	50 (8.8)	38 (6.7)	0.18

*Note:* Italic value indicate statistically significant at *p* < 0.05.

Abbreviation: EF = ejection fraction.

Demographic parameters and detailed baseline characteristics of the study and control groups are shown in Supporting Information S1: Tables 2 and 3. The aged group consisted of 486 patients (252 males [51.9%], in whom a total of 566 EP procedures [2.3% of all procedures in this time period]) were performed. The mean patient age was 82.7 ± 2.5 years and ranged from 80 to 95 years. Four hundred and eighty‐one patients were aged 80−85 years, 73 were aged 85−90 years, 10 were aged 90−95 years, and 2 patients were older than 95 years. Arterial hypertension (*n*: 415, 73.3%) and coronary heart disease (*n*: 215, 38%) were the most common cardiovascular risk factors. Valvular heart disease diseases (*n*: 157, 27.7%) and chronic renal failure (*n*: 129, 22.8%) were common comorbidities. Structural heart diseases (*n*: 98, 17.3%), diabetes mellitus (DM) (*n*: 70, 12.4%), and lung diseases (*n*: 66, 11.7%) were less common. Fifty‐six patients (9.9%) had a previous myocardial infarction, and in 139 patients, an implanted cardiac device was present (24.6%).

A cohort of 480 individuals (358 male [63.3]) who underwent a total of 566 procedures served as a control group (“younger group”). Baseline characteristics of the aged and younger groups were significantly different in terms of patient age (82.7 ± 2.5 vs. 64.1 ± 13.3 years, *p* < 0.001), amount of male gender (44.5% vs. 63.3%, *p* < 0.001), mean LVEF (56.7 ± 13.6% vs*.* 53.9 ± 11.2%, *p* < 0.001), and the number of patients with chronic renal disease (22.8% vs*.* 6.3%, *p* < 0.001) (Table 1).

There was no difference in terms of performed procedures in each group after matching (Table 2). AF/AT ablation was the most commonly performed procedure in the aged group (*n*: 265, 45.2%, 32 [5.7%] of these were cryoballoon ablation procedures), followed by CTI ablation (*n*: 139, 24.6%). The mean age of patients who underwent CTI ablation was 83.0 ± 2.6 and did not differ significantly from the total patient cohort. Ablation of VT or premature ventricular complexes (PVC) were performed in 57 procedures (10.1%; 39 patients [6.9%] with endocardial access and by epicardial access in 1 case [0.2%]). Atrial tachycardia ablation was performed in 19 procedures (3.4%), SVT in 65 procedures (11.5%), and atrioventricular node ablation in 17 procedures (3.0%). LAAC was performed in 12 procedures (2.1%). Femoral vein access was utilized in all cases. Transseptal access to the left atrium or ventricle was obtained in 327 procedures (57.8%). Arterial access and subclavian vein access was utilized in 48 (8.5%) and 83 (14.7%) procedures, respectively.

**Table 2:** Procedural data of the aged and younger groups.

	Aged group	Younger group	
Parameter	*n* (%)	*n* (%)	*p* value
Procedures	566	566	
AF/AT	256 (45.2)	256 (45.2)	1.0
Cryoballoon ablation	32 (5.7)	32 (5.7)	1.0
CTI	139 (24.6)	139 (24.6)	1.0
AVN ablation	17 (3.0)	17 (3.0)	1.0
SVT	66 (11.7)	66 (11.7)	1.0
PVC	17 (3.0)	17 (3.0)	1.0
VT endo	39 (6.9)	35 (6.2)	0.63
VT epi	1 (0.2)	5 (0.9)	0.22
LAAC	12 (2.1)	12 (2.1)	1.0
At least one major complication or death	34 (6.0)	22 (3.9)	0.10
At least one major complication	32 (5.7)	21 (3.5)	0.12
Bleeding at vascular access site	14 (2.5)	13 (2.2)	
Cardiac tamponade	8 (1.4)	5 (0.8)	
Pulmonary embolism	2 (0.4)	0 (0.0)	
Stroke	2 (0.4)	1 (0.2)	
Respiratory failure with need for mechanical ventilation	2 (0.4)	0 (0.0)	
AV block with permanent pacemaker therapy	2 (0.4)	0 (0.0)	
Pneumothorax with drainage	2 (0.4)	0 (0.0)	
Phrenic nerve palsy	1 (0.2)	1 (0.2)	
Gastroparesis	0 (0.0)	1 (0.2)	
Minor complications *n* (%)	31 (5.1)	17 (3.0)	*0.039*
Death	6 (1.1)	1 (0.2)	0.12
In patients with cardiac tamponade	2 (0.5)	0 (0.0)	
In patients with pulmonary embolism	2 (0.5)	0 (0.0)	
In patients with pneumonia	1 (0.2)	0 (0.0)	
In patients with decompensated heart failure	1 (0.2)	1 (0.2)	

*Note:* Italic values indicates statistically significant at *p* < 0.05.

Abbreviations: AF/AT = atrial fibrillation/atrial tachycardia, AV = atrioventricular, AVN = atroventricular node, CTI = cavotricuspid isthmus, LAAC = left atrial appendage closure, PVC = premature ventricular contraction, SVT = supraventricular tachycardia, VT = ventricular tachycardia.

### 3.1 Frequency of Periprocedural Adverse Events and Intrahospital Death in Aged and Younger Individuals

A total of 32 procedures of the aged group were accompanied by the occurrence of at least 1 major complication (5.7% of performed procedures) with a total of 33 major complications (Table 2). Major complications were access site complications resulting in blood transfusion or interventional or surgical treatment in 14 cases, cardiac tamponade in 8 cases, pulmonary embolism in 2 cases, stroke in 2 cases, respiratory failure with need for invasive ventilation in 2 cases, AV block with subsequent permanent pacemaker implantation in 2 cases, pneumothorax treated by drain in 2 cases after subclavian vein access, and phrenic nerve palsy related to catheter ablation in 1 case. Twenty‐one major complications (70% of all major complications) occurred during or after procedures containing transseptal puncture. Minor complications occurred during the hospital stay of 29 procedures (5.1%). Six patients of the aged group died during the hospital stay after the EP procedure (2 cases of cardiac tamponade, 2 pulmonary artery embolism, 1 decompensated left heart failure during VT storm, 1 pneumonia after aspiration during the ablation procedure) with 5 cases which were directly related to procedural‐associated adverse events.

At least one major complication occurred in 21 procedures (3.5%) in the control group. There was 1 case of intrahospital death in the control group which was not directly associated to the EP procedure (a case of decompensated heart failure with cardiogenic shock after the EP procedure). The frequency of adverse events of the study and control groups in relation to the type of performed procedure is shown in Table 3. The frequency of adverse events and intrahospital deaths were numerically higher in the aged group but the difference did not reach statistical significance (*p *= 0.12 for major complications and for intrahospital death, Table 3). There were significantly more procedures with minor complications in the aged group (31 [5.1%] vs. 17 [20%], *p* = 0.039).

**Table 3:** Adverse events in the aged and younger groups.

Type of procedure	*n* patients (aged vs. younger)	Major complications (aged vs. younger)	*p*	Minor complications (aged vs. younger)	*p*	Intrahospital death (aged vs. younger)	*p* value
AF/AT	275 versus 275	14 (5.1) versus 8 (2.9)	0.19	15 (5.5) versus 13 (4.7)	0.69	3 (1.1) versus 0 (0.0)	0.25
CTI	139 versus 139	3 (2.2) versus 3 (2.2)	1.0	7 (5.0) versus 1 (0.7)	0.066	1 (0.7) versus 0 (0.0)	1.0
AVN ablation	17 versus 17	1 (5.9) versus 0 (0.0)	1.0	2 (11.8) versus 0 (0.0)	0.48	0 (0.0) vs 0 (0.0)	1.0
SVT	66 versus 66	2 (3.0) versus 1 (1.4)	0.56	3 (2.0) versus 2 (3.0)	1.0	0 (0.0) versus 0 (0.0)	1.0
Atrial arrhythmias	497 versus 497	20 (4.0) versus 12 (2.4)	0.15	27 (5.4) versus 16 (3.2)	0.12	4 (0.8) versus 0 (0.0)	0.12
PVC	17 versus 17	4 (23.5) versus 2 (8.3)	0.21	2 (11.8) versus 1 (5.9)	1.0	0 (0.0) versus 0 (0.0)	1.0
VT endo	39 versus 35	6 (15.4) versus 4 (10.5)	0.40	1 (2.6) versus 0 (0.0)	1.0	1 (2.6) versus 1 (2.9)	1.0
VT epi	1 versus 5	0 (0.0) versus 1 (20)	1.0	0 (0.0) versus 0 (0.0)	1.0	0 (0.0) versus 0 (0.0)	1.0
Ventricular arrhythmias	57 versus 57	10 (17.5) versus 7 (10.4)	0.43	3 (5.3) versus 1 (1.8)	0.62	1 (1.8) versus 1 (1.8)	1.0
LAAC	12 versus 12	2 (16.7) versus 1 (8.3)	0.54	1 (8.3) versus 1 (8.3)	1.0	1 (8.3) versus 0 (0.0)	1.0
All procedures	566 versus 566	32 (5.8) versus 21 (3.5)	0.12	31 (5.1) versus 17 (3.20	*0.039*	6 (1.1) versus 1 (0.2)	0.12

*Note:* Italic value indicates statistically significant at *p* < 0.05.

Abbreviations: AF/AT = atrial fibrillation/atrial tachycardia, AVN = atroventricular node, CTI = cavotricuspid isthmus, LAAC = left atrial appendage closure, PVC = premature ventricular contraction, SVT = supraventricular tachycardia, VT = ventricular tachycardia.

### 3.2 Frequency of Adverse Events in Relation to Performed EP Procedure in the Aged Group

**Figure 1:**
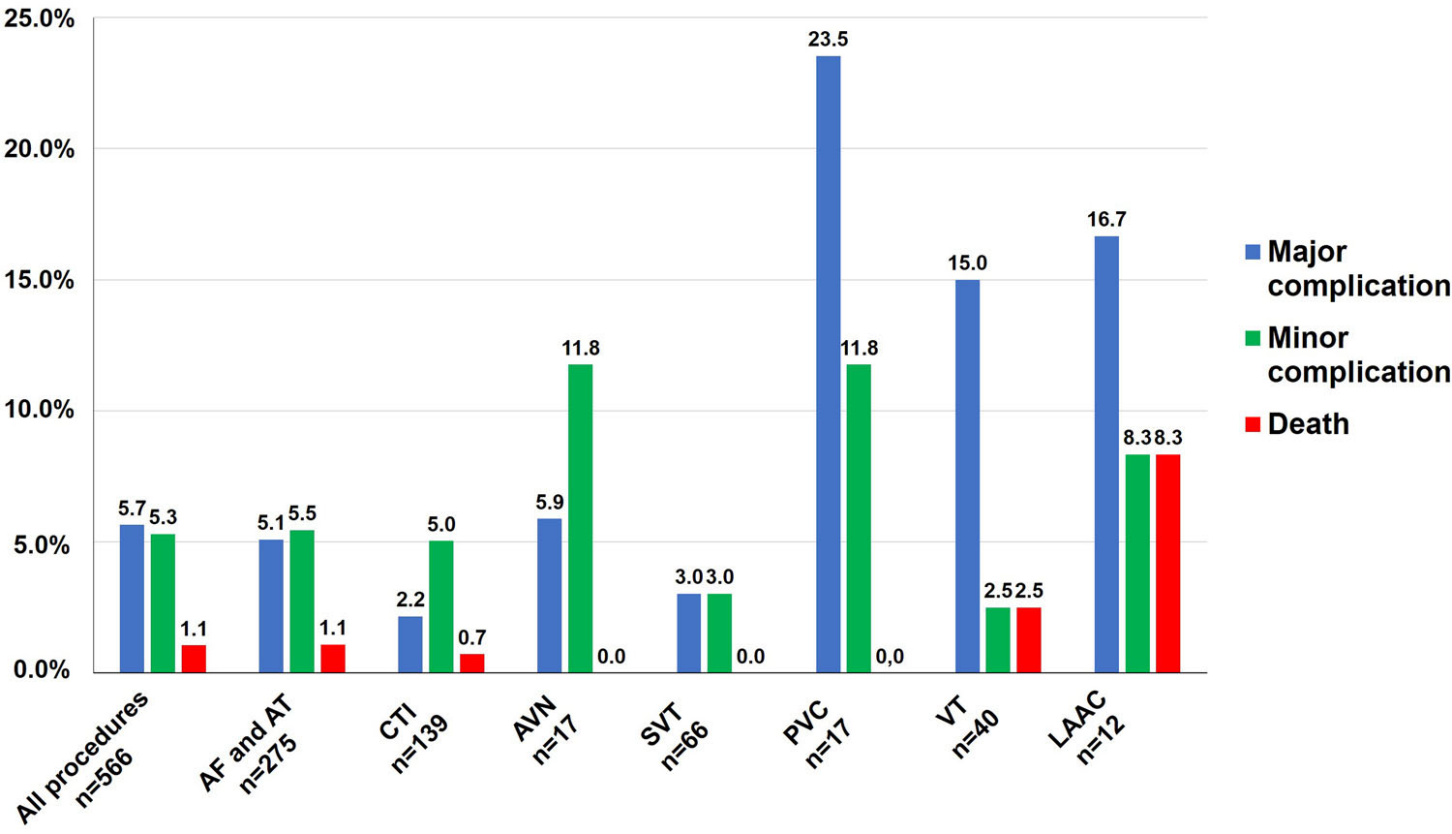
Adverse events in the aged group stratified by type of procedure. AF/AT = atrial fibrillation/atrial tachycardia, AVN = atroventricular node, CTI = cavotricuspid isthmus, LAAC = left atrial appendage closure, PVC = premature ventricular contraction, SVT = supraventricular tachycardia, VT = ventricular tachycardia.

The frequency of major and minor complications and intrahospital death in relation to the performed procedure types is shown in Figure 1. During atrial arrhythmia treatment, major complications occurred in 2.2%−5.9% of procedures (AF/AT 5.1% [Cryoballoon ablation 3.1%], CTI ablation 2.2%, AVN ablation 5.9%, SVT 3.0%), while major complications during treatment of ventricular arrhythmias occurred in 23.5% of PVC procedures and 15.0% of endo‐ or epicardial VT procedures. LAAC procedures had a frequency of major complications of 16.7%. The frequency of intrahospital death for the most common types of procedures was 1.1% for AF/AT ablation and 0.7% for CTI ablation and ranged from 0.0% for AVN/SVT/PVC ablation over 2.5% for VT ablation to 8.3% for LAAC procedures.

### 3.3 Frequency of Adverse Events in Relation to Patient Age in the Aged Group

**Figure 2:**
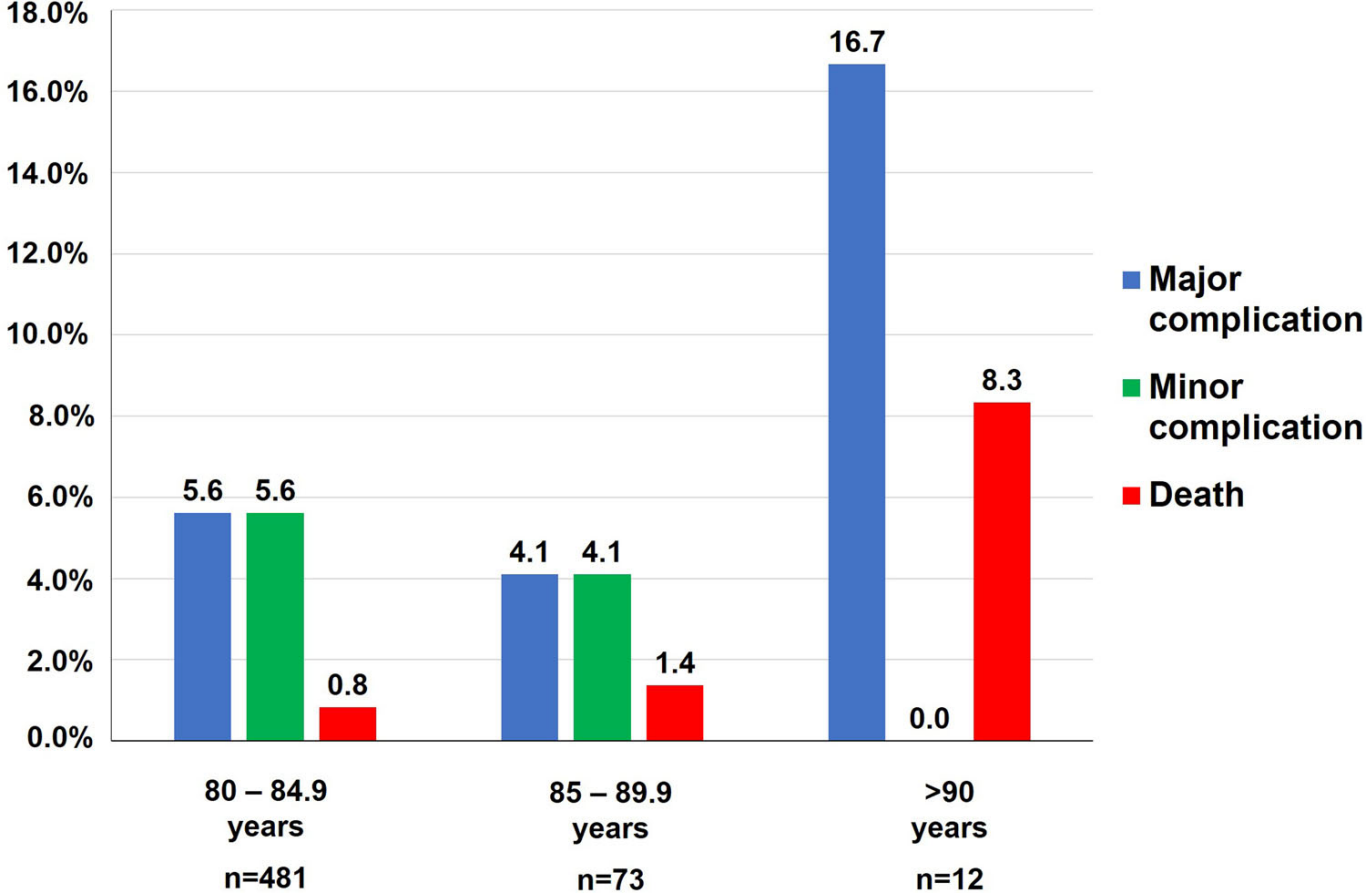
Adverse events in the aged group stratified by age.

Analysis of the frequency of adverse events in age subgroups is presented in Figure 2. The frequency of major complications in procedures of patients aged from 80 to 84.9 years was 5.6%, in procedures of patients aged 85−89 years 4.1% and in procedures of patients above 90 years of age 16.7%, respectively. Intrahospital death occurred during the hospital stay of 0.8% of procedures in patients aged 80−84.9 years (2 cases of cardiac tamponade, 2 pulmonary embolisms), 1.4% of procedures of patients aged 85−89 years of age (1/73, a patient with VT storm and subsequent cardiac output failure), and 8.3% patients (1/12 patients above 90 years, a 93‐years old patient with intraprocedural hemoptysis and subsequent aspiration pneumonia during a cryoballoon PVI).

### 3.4 Frequency of Early Recurrence and Adverse Events Unrelated to Interventional Procedures in the Aged Group

**Figure 3:**
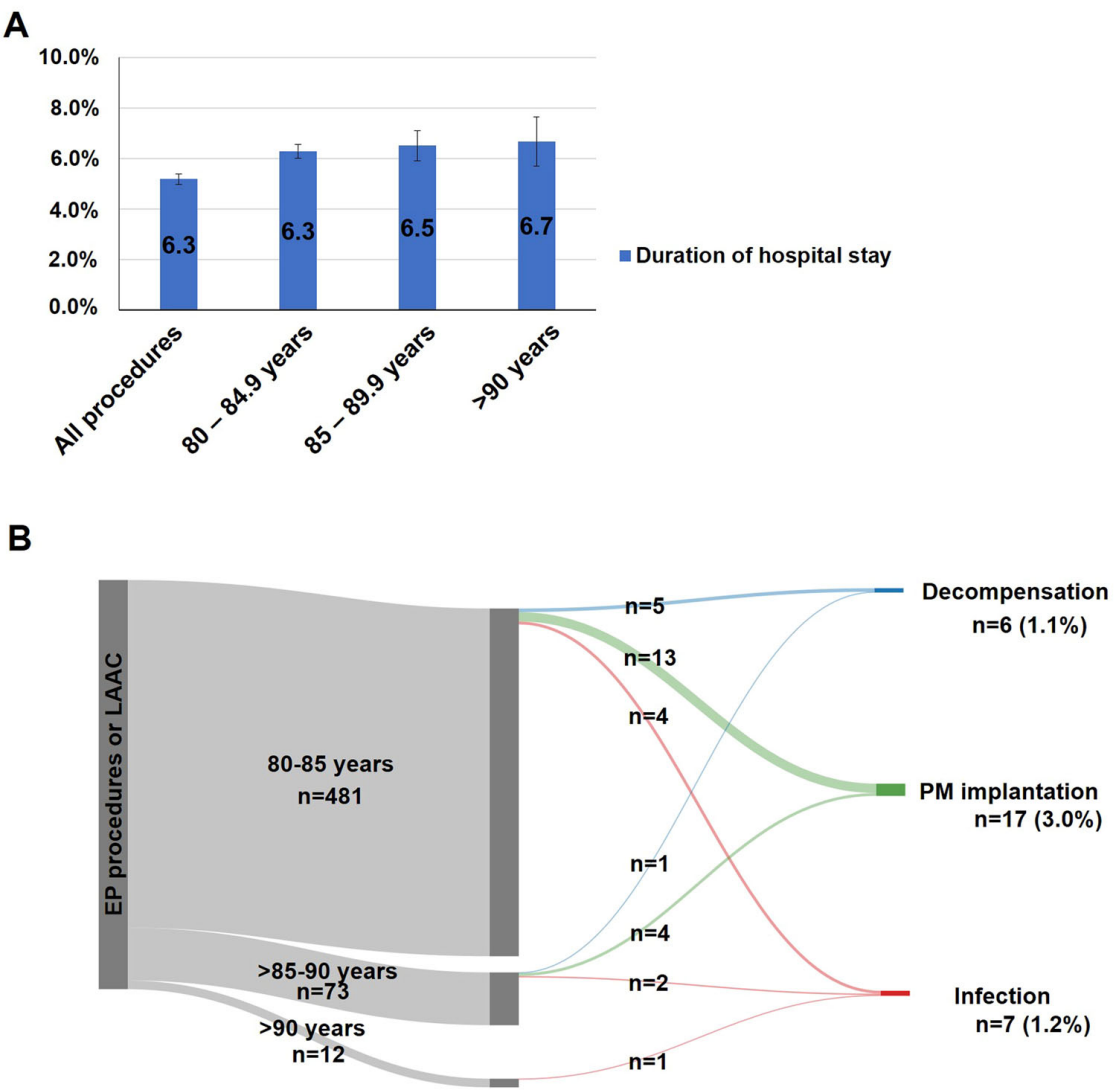
(A) Mean duration of hospital stay stratified by age in the aged group. (B) Periprocedural adverse events not related to the procedure in the aged group. EP = electrophysiologic, LAAC = left atrial appendage closure, PM = pacemaker.

In‐hospital arrhythmia recurrence occurred in 84 patients (14.8% of procedures) in the aged group. The mean duration of hospital stays in the aged group was 6.3 ± 5.8 days and was comparable in different age groups and is displayed in Figure 3A. There were 30 adverse events in the aged group which were not clearly related to the EP procedure. There were seven infections during hospital stay, 6 cases of decompensated heart failure and 17 pacemaker implantations due to various reasons which were not a complication associated to catheter ablation (Figure 3B).

### 3.5 Logistic Regression Model on Risk Factors for Complications and Intrahospital Death

**Figure 4:**
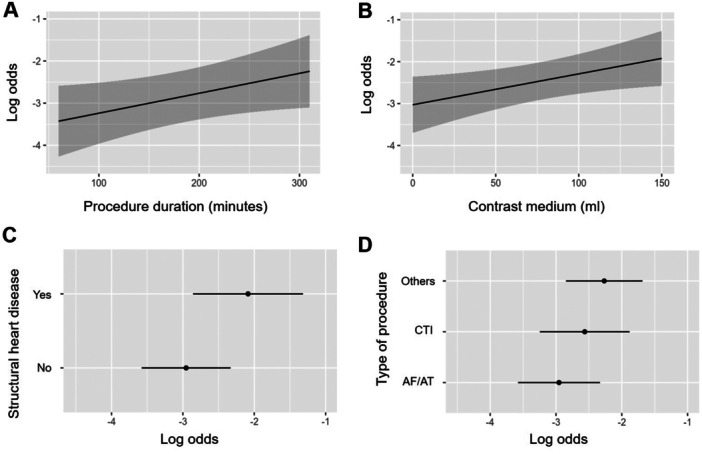
Logarithmic odds in relation to procedure duration (A), contrast medium volume (B), presence/absence of structural heart disease (C), and different types of procedures (D).

The following parameters were tested in a logistic regression model on parameters of the aged group: Type of ablation (AF/AT, CTI, other), LVEF, renal failure, transseptal puncture, anticoagulation, antiplatelet therapy, contrast medium volume, age, number of previous ablations, DM, structural heart disease, procedure duration. Logistic regression revealed presence of structural heart disease (odds ratio 2.38, *p* = 0.0067), procedure duration (odds ratio 1.98, *p* = 0.0483), contrast medium volume (odds ratio 2.22, *p* = 0.0262), and type of procedure (other than AF/AT and CTI) (odds ratio 1.96, *p* = 0.0499) as factors with significant impact on the occurrence of complications and death in the aged group (Table 4). Figure 4 shows logarithmic odds in relation to procedure duration (A) and contrast medium volume (B) as well as for the presence/absence of structural heart disease (C) and different types of procedures (D).

**Table 4:** Logistic regression analysis for prediction of periprocedural complications.

Parameter	*ß*	SE	Wald *Z*	Odds ratio	Lower 0.95	Upper 0.95	*p* value
Presence of structural heart disease	0.8677	0.3199	2.71	2.38	1.27	4.46	*0.0067*
Procedure duration	0.0047	0.0024	1.98	1.46	1.00	2.12	*0.0483*
Contrast medium volume	0.0074	0.0033	2.22	2.09	1.09	4.00	*0.0262*
Type of procedure: CTI^a^	0.3911	0.4610	0.85	1.48	0.60	3.65	0.3962
Type of procedure: Other type than CTI or AF/AT^a^	0.6872	0.3505	1.96	1.99	1.00	3.95	*0.0499*

*Note:* Five selected parameters (four significant variables and type of procedure: CTI) are displayed. All other parameters which were tested did not show significant association of the tested outcome. The odds ratio describes the likelihood to develop an adverse event. Italic values indicate statistically significant at *p* < 0.05.

Abbreviations: AF/AT = atrial fibrillation/atrial tachycardia, CTI = cavotricuspid isthmus.

^a^ AF/AT was set as a reference value.

## 4 Discussion

We report on outcomes of interventional EP procedures in a large patient sample of octogenarians and nonagenarians. To the best of our knowledge, this study is the analysis of the largest published patient sample so far. We found that interventional EP procedures were accompanied by significantly comparable rates of complications and intrahospital deaths in aged versus younger patients across a wide spectrum of procedures. Nevertheless, there were significantly more minor complications and numerically more major complications and intrahospital deaths in aged patients as compared to younger controls.

### 4.1 Invasive EP Procedures in the Elderly

Until today, the majority of patients who are studied in clinical trials on catheter ablation of cardiac arrhythmias or LAAC are below 80 years of age. In a recent EHRA survey age was one of the most common parameters which was taken into account for patient selection for AF ablation [4]. The 2017 HRS/EHRA/ECAS7APHRS/SOLAECE consensus document on AF ablation stated that AF ablation can be performed in aged patients (class IIa, level of evidence B recommendation) but noted that perioperative complications may be higher and arrhythmia recurrence may be more common as in younger patients [1]. There is no guideline recommendation on invasive procedures for ventricular arrhythmias in aged patients until today [2]. Outcomes of invasive EP procedures in aged patients were investigated in smaller, non‐randomized studies. Metzner et al. investigated outcomes of patients over 75 years of age who underwent radiofrequency (RF) AF ablation [5]. In this study, major complications were noted in 5.8% of procedures [5]. In a study by Moser et al., 227 patients > 75 years, who underwent RF AF ablation, were compared to 4222 patients < 75 years [6]. In this study, the overall rate of complications was comparable between groups but significantly more periprocedural strokes occurred in aged patients [6]. Cryoballoon ablation was studied in several smaller trials. Other studies investigated patients above and below 75 years of age who underwent CB ablation [7, 8] and found that periprocedural safety as well as arrhythmia‐free survival was comparable for aged and younger patients. In a sub‐analysis from the CABANA trial, no age‐related increase of periprocedural major adverse events was observed, but the previously modest prognostic effects of catheter ablation were even ameliorated in aged individuals [9]. In a recent meta‐analysis, a total of 110 606 patients including 8009 elderly individuals > 80 years of age were analyzed [10]. The study found comparable effectiveness in terms of arrhythmia‐free survival but a significantly higher rate of periprocedural adverse events in aged patients [10]. Interestingly, higher complications were only found in patients who underwent RF ablation, while CB procedures resulted in comparable rates of cardiac tamponades and phrenic nerve injuries in younger and elderly patients [10]. Of note, only two studies which were included in this meta‐analysis included patients > 80 years of age. A recent study which used the National Inpatient Sample database cases investigated 3482 patients > 80 years who underwent AF ablation and compared them to 82 637 younger patients [11]. This study found significantly higher rates of periprocedural complications (16.2% vs. 9.8% overall complications, 3.6% vs. 2.8% major complications) while the periprocedural mortality was similar [11]. Other smaller studies found no difference in procedural safety in patients > 80 years and younger patients who underwent RF or CB ablation [12, 13, 14, 15, 16, 17, 18, 19]. Another recent multi‐center study from 13 centers in the UK, France, and Switzerland compared the results of AF ablation in 473 octogenarians and younger patients [20]. In this study, octogenarians suffered from significantly more frequent procedural complications (11.4% vs. 7.0%) as well as significantly more frequent arrhythmia relapse after a median of 281/354 days of follow‐up (27.7% vs. 23.5%) [20]. Multivariate analysis found age to be a significant independent predictor of arrhythmia recurrence [20].

There are only a few systematic studies on ventricular arrhythmia ablation in aged patients. A study by Vakil et al. investigated catheter ablation for ventricular arrhythmia treatment in the presence of structural heart disease in aged patients > 70 years (*n* = 681) and younger controls (*n* = 1368) [21]. The study found comparable periprocedural complications and acute and long‐term effectiveness of arrhythmia suppression but procedures were accompanied by more frequent intrahospital deaths in the aged patient group [20]. Another study reported on patients > 75 years who underwent ablation for VT in the presence of structural heart disease and found comparable frequencies of periprocedural complications and deaths as compared to younger patients [22]. Two studies assessed outcomes of 11 and 54 octogenarians who underwent ventricular arrhythmia ablation [23, 24]. The rates of major complications were 37% (3/11) and 18% (vs. 2% in a younger control group), respectively [23, 24]. Additionally, one patient died in the former study which was not procedure‐related. Of note, 1/11 patients (9.9%) from the former and 28% of patients from the latter study received further ICD therapies after catheter ablation [23, 24]. A study by Darma et al. compared patients with structural heart disease aged 75−79 years with octogenarians who underwent VT ablation [25]. The study found statistically comparable frequencies of periprocedural major complications and intrahospital deaths [24]. Nevertheless, with respect to small patient numbers, the relatively high rates of events (16% major complications and 59% deaths in octogenarians) are remarkable [25]. Nevertheless, many patients suffered from electrical storms before catheter ablation was performed, and high rates of complications and mortality also in younger patients were described for this condition. A recent meta‐analysis concluded that VT ablation in aged patients is effective, but is accompanied by increased rates of periprocedural complications and mortality as compared to younger patients [26].

Percutaneous LAAC has become a routine procedure today. Several studies investigated LAAC in patients with advanced age. Recently, two large US hospital registry analyses found that LAAC was accompanied by higher rates of periprocedural complications and death in patients > 80 years as compared to younger individuals [27, 28]. Additionally, aged patients had higher rates of postprocedure hospital readmissions [28]. Our patient cohort was too small to enable the demonstration of a statistically significant difference between study outcomes. Nevertheless, in line with the above‐mentioned studies, we found numerically higher rates of periprocedural major adverse events and death. Importantly, Sanjoy et al. retrospectively investigated a total of 6779 cases from the National In‐hospital Sample database of patients who underwent LAAC between 2015 and 2018 in the United States [27]. There were 2371 cases of patients > 80 years of age, representing 35% of patients who underwent LAAC in this time period [27]. This high number of aged patients who underwent LAAC demonstrates the importance of such studies which investigate interventional procedures in elderly patients.

To the best of our knowledge, this study investigated the largest patient cohort of patients > 80 years who underwent a broad spectrum of invasive EP procedures. A strength of our analysis is that we also included patients who underwent procedures for other arrhythmias besides AF as well as LAAC procedures. Compared to the above‐mentioned studies, we found comparable rates of periprocedural complications in our cohort of aged patients. Of note, nonagenarians are underreported until now and no comparison to other studies can be made. We found a trend toward more frequent complications in aged patients when compared to a control group of patients which had a mean age of 63 years. Additionally, a trend toward more frequent intrahospital deaths in elderly patients was noted. This trend was not only seen in the overall cohort but also for patients who underwent invasive atrial arrhythmia treatment. Previous studies on AF ablation did not find increased rates of intrahospital mortality, but a comparable finding was reported on outcomes of VT ablations in octogenarians [25]. Interestingly, albeit we observed relatively high rates of major and minor complications in aged patients who underwent ventricular arrhythmia ablation, we did not observe statistically significant more intrahospital deaths. Nevertheless, our results are limited by the relatively low number of investigated patients.

The relatively high intrahospital mortality in our aged group reflects the impact of comorbidities, which are typically found in elderly, on patient outcomes. Three of six deaths after EP procedures were directly related to intraprocedural complications such as cardiac tamponades and intraprocedural hemoptysis leading to pneumonia. These complications have a higher likelihood not to result in lethal outcomes in individuals without marked comorbidities. There were also 2 cases of pulmonary embolism which occurred after EP procedures. In recent days, the duration of hospitalization for EP procedures may be shortened since vascular closure techniques have been improved utilizing sutures and closure devices. Additionally, EP procedures are frequently performed with overnight stays or with a same‐day discharge strategy. If these changes would have a beneficial or negative effect on outcomes of elderly patients is unknown and needs further investigation.

### 4.2 How to Identify Aged Patients at Risk for Adverse Events During Invasive EP Procedures

We found four different factors which were associated with the occurrence of adverse events. Our analysis shows that prolonged and contrast medium intensive procedures may prone aged patients to suffer adverse events. Patients with known structural heart disease are at a higher risk for the development of complications. Finally, more complex procedures like ablation of ventricular arrhythmias bear higher risks of complications as compared to less complex procedures. Our analysis set AF/AT ablations as a baseline level and compared CTI block and other procedures to outcomes of AF/AT ablations. A limitation of our analysis is that AF/AT ablations itself may already be complex procedures in aged patients. Importantly, patients in our study cohort were only a small amount of all patients who underwent EP procedures in our center between 2005 and 2017 (about 2% of the total amount of performed procedures in the center). Even in this selected population of aged patients, relatively high rates of adverse events and deaths were observed. Our data point to the necessity of strict patient selection if interventional treatment is attempted if patients have marked comorbidities, such as structural heart disease or renal failure

We found a relatively high rate of patients who underwent pacemaker implantation during the hospital stay, albeit only cases followed iatrogenic AV block after slow pathway ablation. In the remaining cases, patients showed bradycardias which were masked by concomitant atrial arrhythmias and became obvious after successful rhythm control via invasive treatment. This fact in mind a rate control strategy utilizing a “pace and ablate” approach might be a more favorable option as compared to interventional treatment aiming at rhythm control in a subset of patients.

## 5 Limitations

This was a retrospective, single‐center analysis with its typical limitations. Aged patients which were selected to be suitable to undergo invasive procedures may not fit to a general population of aged individuals. This study does not report on long‐term outcomes of EP procedures. The number of patients who underwent LAAC and who were above 90 years of age was limited which makes it difficult to draw conclusions for very aged collectives. Our patient cohort had a relatively long duration of hospital stay, which may be related to clinical practice during the timespan of the study.

## 6 Conclusion

Invasive EP treatments in octogenarians and nonagenarians are feasible, however a higher incidence of minor periprocedural complications and trend toward more severe complications and intrahospital fatalities were observed compared to younger patients. Our findings support an individual risk‐benefit assessment for elderly individuals before EP procedures are conducted (Central Illustrations 1).

**Central Illustrations 1:**
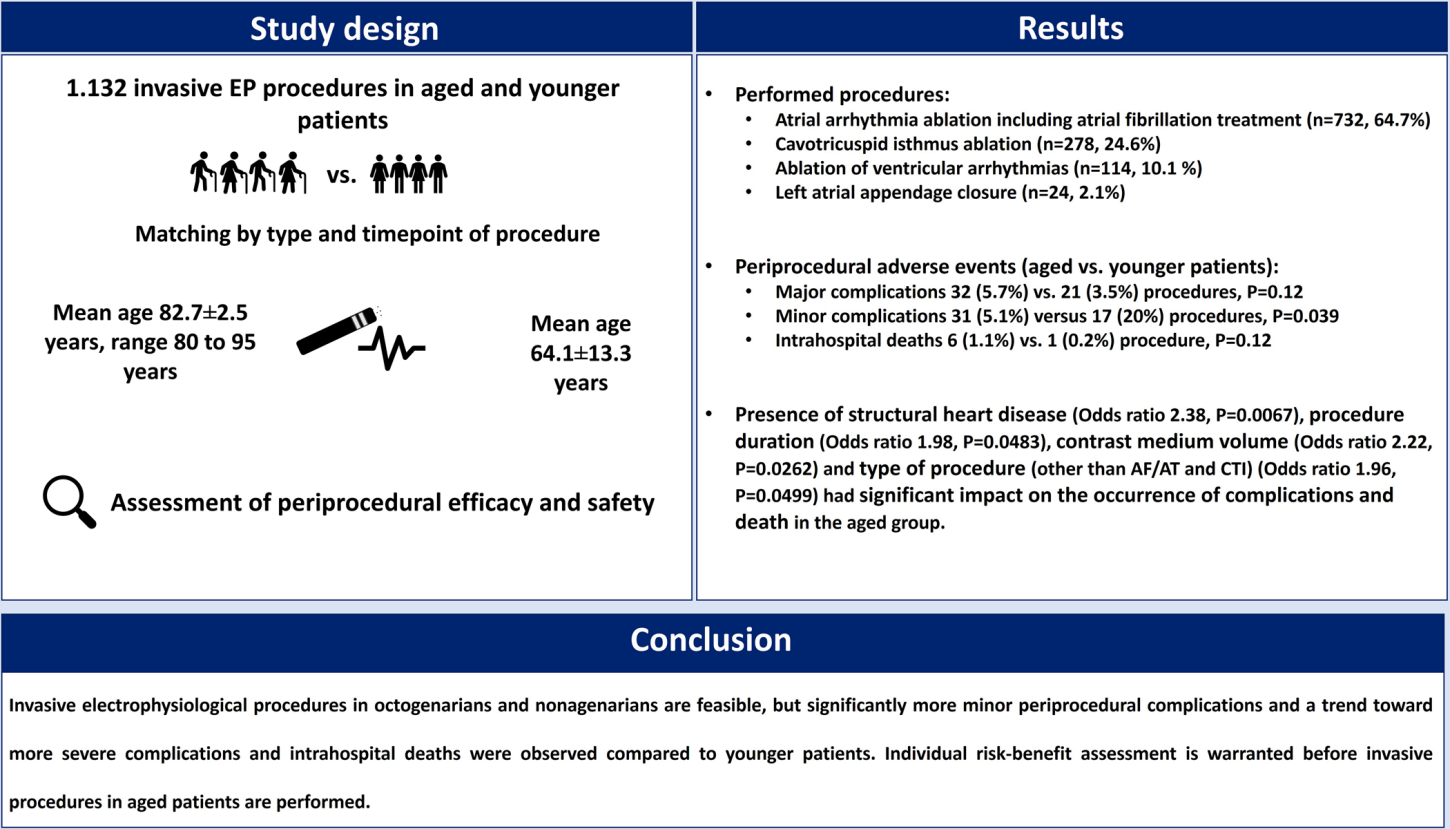
Analysis of invasive electrophysiological procedures in aged patients and a control group of younger individuals.

## Conflicts of Interest

The authors declare no conflicts of interest.

## Supporting information

Supplemental_material.

## Data Availability

The data that support the findings of this study are available from the corresponding author upon reasonable request.
