# Supplementary material for: Periprocedural Safety of Interventional Electrophysiological Procedures in Octogenarians and Nonagenarians
Source: J Cardiovasc Electrophysiol. 2025 May 26;36(8):1721–32. doi: 10.1111/jce.16689 (PMC12337617; doi:10.1111/jce.16689)
Supplement: Supplementary file 1 — Supplemental_material. [file JCE-36-1721-s001.docx]

**Supplemental table 1** Performed procedures from 2005 to 2017

|  | **All procedures** | **SVT/EP study** | **AF** | **AT (RA, LA)** | **CTI** | **AVN** | **VT endo** | **VT epi** | **LAAC** | **Procedures in patients >80 years** | **% of procedures** |
| --- | --- | --- | --- | --- | --- | --- | --- | --- | --- | --- | --- |
| **2005** | 1128 | 414 | 469 | 64 | 163 | 1 | 87 | 7 | 0 | 9 | 0.8 |
| **2006** | 1413 | 393 | 859 | 56 | 142 | 3 | 73 | 11 | 2 | 14 | 1.0 |
| **2007** | 1594 | 369 | 1038 | 66 | 113 | 3 | 79 | 26 | 7 | 27 | 1.7 |
| **2008** | 1688 | 520 | 934 | 141 | 159 | 3 | 100 | 29 | 4 | 25 | 1.5 |
| **2009** | 1824 | 563 | 1105 | 107 | 143 | 3 | 122 | 7 | 4 | 22 | 1.2 |
| **2010** | 1977 | 357 | 1235 | 111 | 168 | 5 | 134 | 25 | 29 | 33 | 1.7 |
| **2011** | 2017 | 335 | 1350 | 111 | 104 | 5 | 140 | 33 | 39 | 41 | 2.0 |
| **2012** | 2005 | 322 | 1323 | 98 | 119 | 2 | 177 | 24 | 53 | 47 | 2.3 |
| **2013** | 2170 | 356 | 1349 | 130 | 119 | 8 | 204 | 28 | 66 | 52 | 2.4 |
| **2014** | 2209 | 240 | 1505 | 94 | 99 | 11 | 233 | 16 | 69 | 41 | 1.9 |
| **2015** | 2139 | 283 | 1338 | 103 | 168 | 18 | 224 | 19 | 66 | 53 | 2.5 |
| **2016** | 2125 | 272 | 1299 | 122 | 140 | 23 | 250 | 27 | 73 | 104 | 4.9 |
| **2017** | 2159 | 287 | 1368 | 119 | 136 | 11 | 220 | 17 | 46 | 98 | 4.5 |
| **Total** | 24448 | 4711 | 15172 | 1322 | 1773 | 96 | 2043 | 269 | 458 | 566 | 2.3 |

AF/AT=Atrial fibrillation/atrial tachycardia, RA=Right atrial, LA=Left atrial, CTI=Cavotricuspid isthmus, AVN=Atroventricular node, SVT=Supraventricular tachycardia, PVC=Premature ventricular contraction, VT=Ventricular tachycardia, LAAC=Left atrial appendage closure.

**Supplemental table 2** Demographical parameters of the aged group

| **Parameter** | **n (%) or mean±SD** |
| --- | --- |
| **Patients/procedures** | 486/566 |
| **Age (years)** | 82.7±2.5 |
| **Age range (years)** | 80.0-95.3 |
| **80-85 years, n (%)** | 481 |
| **85-90 years, n (%)** | 73 |
| **90-95 years, n (%)** | 10 |
| **>95 years, n (%)** | 2 |

SD=standard deviation.

**Supplemental table 3** Baseline parameters of study patients

| **Parameter** | **n (%) or mean±SD** | **Parameter** | **n (%) or mean±SD** |
| --- | --- | --- | --- |
| **patients/procedures** | 486/566 | **Antiplatelet therapy , n (%)** | 134 (23.7) |
| **Body size (cm)** | 169.2±13.6 | **ASS, n (%)** | 103 (18.2) |
| **Body weight (kg)** | 75.5±12.1 | **Clopidogrel, n (%)** | 15 (2.7) |
| **Body mass index** | 26.6±8.6 | **Ticagrelor, n (%)** | 1 (0.2) |
| **Male, n (%)** | 252 (51.9) | **DAPT, n (%)** | 15 (2.7) |
| **Cardiac comorbidities** |  | **ASS+Clopidogrel, n (%)** | 13 (2.3) |
| **Structural heart disease, n (%)** | 98 (17.3) | **ASS+Ticagrelor, n (%)** | 2 (0.4) |
| **Mean EF (%)** | 56.7±13.6 |  |  |
| **EF<50 %, n (%)** | 31 (5.5) | **Oral anticoagulation, n (%)** | 433 (76.5) |
| **EF<30 %, n (%)** | 12 (2.1) | **Vitamin K antagonist, n (%)** | 183 (32.3) |
| **Implantable cardiac device, n (%)** | 139 (24.6) | **DOAC, n (%)** | 250 (44.2) |
| **Coronary artery disease, n (%)** | 215 (38.0) | **Rivaroxaban, n (%)** | 109 (19.3) |
| **Previous myocardial infarction, n (%)** | 56 (9.9) | **Dabigatran, n (%)** | 28 (5.0) |
| **Valvular heart disease, n (%)** | 157 (27.7) |  |  |
| **Other comorbidities** |  | **Antiarrhythmic drugs, n (%)** | 283 (50.0) |
| **Arterial hypertension, n (%)** | 415 (73.3) | **Flecainide, n (%)** | 88 (15.6) |
| **Diabetes Mellitus, n (%)** | 70 (12.4) | **Amiodarone, n (%)** | 179 (31.6) |
| **Kidney failure, n (%)** | 129 (22.8) | **Solatol, n (%)** | 6 (1.1) |
| **Lung disease, n (%)** | 66 (11.7) | **Dronedarone, n (%)** | 9 (1.69 |
| **Previous stroke or TIA, n (%)** | 50 (8.8) | **Mexiletine, n (%)** | 1 (0.2) |

EF=Ejection fraction, DAPT=Dual antiplatelet therapy, DOAC=Direct oral anticoagulation, SD=standard deviation.

**Supplemental table 4** Detailed procedural data of the aged group

| **Parameter** | **n (%) or mean±SD** |
| --- | --- |
| **Procedure duration (min)** | 160.0±78.7 |
| **Fluoroscopy duration (min)** | 15.7±35.9 |
| **Radiation dosage (cGy*cm^2^)** | 1460±1738 |
| **Contrast medium volume (ml)** | 39.7±47.9 |
| **Early arrhythmia recurrence during hospital stay (n)** | 84 (14.8) |

SD=standard deviation.

**Supplemental table 5** Periprocedural adverse events in relation to timepoint of invasive EP procedure

| **Timespan** | **Category** | **n patients** | **Major compliation** | **P** | **Minor complication** | **P** | **Intrahospital death** | **P** |
| --- | --- | --- | --- | --- | --- | --- | --- | --- |
| **2005-2009** | **Aged group** | 97 | 3 (3.1%) | 0.49 | 7 (7.2%) | 0.17 | 0 (0.0%) | 1.0 |
|  | **Younger group** | 97 | 6 (6.2%) |  | 2 (2.1%) |  | 1 (1.0%) |  |
|  | | | | | | | | |
| **2010-2013** | **Aged group** | 173 | 11 (6.4%) | 0.0525 | 15 (8.7%) | 0.0694 | 2 (1.2%) | 1.0 |
|  | **Younger group** | 173 | 3 (1.7%) |  | 6 (3.5%) |  | 0 (0.0%) |  |
|  | | | | | | | | |
| **2014-2017** | **Aged group** | 296 | 18 (6.1%) | 0.25 | 8 (2.7%) | 0.81 | 4 (1.4%) | *<0.001* |
|  | **Younger group** | 296 | 11 (3.7%) |  | 10 (3.4%) |  | 0 (0.0%) |  |
